# Relationship between [^18^F]FDG PET/CT and metabolomics in patients with colorectal cancer

**DOI:** 10.1007/s11306-022-01952-1

**Published:** 2022-11-11

**Authors:** Masashi Imajo, Takashi Norikane, Yuka Yamamoto, Yukito Maeda, Kaori Saitoh, Keiko Kato, Tomoyoshi Soga, Keiichi Okano, Yoshihiro Nishiyama

**Affiliations:** 1 Department of Radiology, Faculty of Medicine, Kagawa University, 1750-1 Ikenobe, Miki-Cho, Kita-Gun, Kagawa 761-0793 Japan; 2 Department of Clinical Radiology, Kagawa University Hospital, Miki-Cho, Kagawa Japan; 3 Institute for Advanced Biosciences, Keio University, Tsuruoka, Yamagata Japan; 4 Department of Gastroenterological Surgery, Faculty of Medicine, Kagawa University, Miki-Cho, Kagawa Japan

**Keywords:** FDG, PET/CT, Colorectal cancer, Metabolome

## Abstract

**Introduction:**

Advances in metabolomics have significantly improved cancer detection, diagnosis, treatment, and prognosis.

**Objectives:**

To investigate the relationship between metabolic tumor volume (MTV) using 2-deoxy-2-[^18^F]fluoro-D-glucose (FDG) positron emission tomography (PET)/ computed tomography (CT) and metabolomics data in patients with colorectal cancer (CRC).

**Methods:**

The metabolome in tumor tissues was analyzed using capillary electrophoresis time-of-flight mass spectrometry in 33 patients with newly diagnosed CRC who underwent FDG PET/CT before treatment and had tumor tissue post-surgery. Based on the FDG PET data, MTV was calculated and was dichotomized according to the median value, and tumors were divided into low-MTV and high-MTV tumors. Metabolomics data were compared between the low-MTV and high-MTV tumors.

**Results:**

The levels of most glycolysis-related metabolites were not different between low-MTV and high-MTV tumors. The level of component of the initial part of the tricarboxylic acid (TCA) cycle, citrate, was significantly lower in the high-MTV tumor than in the low-MTV tumor. The TCA intermediate succinate level was significantly higher in the high-MTV tumor than in the low-MTV tumor. In contrast, the TCA intermediate fumarate level was significantly lower in the high-MTV tumor than in the low-MTV tumor. The levels of many amino acids were significantly higher in the high-MTV tumor than in the low-MTV tumor.

**Conclusions:**

Although preliminary, these results suggest that tumors with high FDG metabolism in CRC may obtain more energy by using a reverse reaction of the TCA cycle and amino-acid metabolism. However, further research is required to clarify this relationship.

## Introduction

Colorectal cancer (CRC) is the third most common cancer and the second most common cause of cancer-related deaths worldwide (Sung et al., 2021). Although advances in multimodality treatment have significantly improved the outcomes for patients with CRC, the prognosis for advanced-stage patients remains poor (Chahine et al., 2019). Positron emission tomography (PET) with 2-deoxy-2-[^18^F]fluoro-D-glucose (FDG) is a useful metabolic imaging modality for staging, re-staging, and assessing the treatment response in CRC cases (Akin et al., 2020). The most commonly used semi-quantitative PET parameter is the standardized uptake value (SUV). Owing to its simplicity, the maximum SUV (SUVmax) within a lesion is mainly used clinically to represent the intensity of radioactivity within the lesion. Currently, volume-based parameters such as the metabolic tumor volume (MTV), are also useful (Larson et al., 1999). The volumetric parameters reflect the metabolic activity in the entire lesion. In contrast, SUVmax reflects that of only a single voxel.

Recently, advances in ‘-omics’ technologies, such as genomics, transcriptomics, proteomics, metabolomics, and the combination of these technologies, have significantly improved cancer detection, diagnosis, treatment, and prognosis (Farid & Morris-Stiff, 2015). Most cancer cells predominantly generate energy by glycolysis rather than by oxidative phosphorylation via the tricarboxylic acid (TCA) cycle, even in the presence of an adequate oxygen supply (Warburg effect) (Warburg, 1956). However, there are few studies on the direct measurement of global metabolites in clinical tumor tissues (Chan et al., 2009; Denkert et al., 2008; Hirayama et al., 2009). Hirayama et al. investigated the differences in metabolomics between normal and tumor tissues in patients with colon cancer. They showed that the lactate and amino acid levels in tumor tissues were higher than those in normal tissues (Hirayama et al., 2009). However, to the best of our knowledge, no studies to date have reported the relationship between metabolomics and FDG PET/ computed tomography (CT) in patients with CRC.

This prompted us to investigate the relationship between MTV using FDG PET/CT and metabolomics data in patients with newly diagnosed CRC.

## Materials and methods

### Patients

This retrospective research protocol was approved by our institutional ethics review committee, and the requirement for obtaining informed consent was waived. From July 2011 to April 2012, 33 patients (20 men and 13 women; mean age, 67 years; age range, 39–95 years) with newly diagnosed colorectal adenocarcinoma who underwent FDG PET/CT prior to treatment and had tumor tissue after surgery were included in the study. Two patients had two primary tumors each. Therefore, 35 tumors (26 colon and 9 rectal cancers) were analyzed. According to the Union for International Cancer Control tumor-node-metastasis (TNM) classification (eighth edition), 8 patients were in stage I, 10 in stage II, 10 in stage III, and 5 in stage IV.

### Metabolome analysis

#### Instrumentation

All capillary electrophoresis time-of-flight mass spectrometry (CE-TOFMS) experiments were performed using Agilent 7100 CE capillary electrophoresis (Agilent Technologies, Waldbronn, Germany), the Agilent 6230 LC/MSD TOF system (Agilent Technologies, Palo Alto, CA, USA), an Agilent1100 series binary HPLC pump, and the G1603A Agilent CE-MS adapter- and G1607A Agilent CE-ESI–MS sprayer kit. For anionic metabolite analysis, the original Agilent stainless electrospray ionization (ESI) needle was replaced with an Agilent G7100-60,041 platinum ESI needle (Soga et al., 2009). System control and data acquisition were performed using the Agilent MassHunter Workstation, and data analysis was performed using Keio MasterHands software.

#### Cationic metabolite analysis

Separations were carried out in a fused silica capillary (50 mm i.d. × 100 cm total length) filled with 1 M formic acid as the electrolyte (Soga et al., 2003, 2006). Approximately 5 nl of the sample solution were injected at 50 mbar for 5 s, and 30 kV of voltage was applied. The capillary temperature was maintained at 20 °C and the sample tray was cooled below 5 °C. Methanol–water (50% v/v) containing 0.01 mM Hexakis(2,2-difluoroethoxy)phosphazene was delivered as the sheath liquid at 10 μl/min. ESI-TOFMS was conducted in the positive ion mode, and the capillary voltage was set at 4000 V. A flow rate of heated dry nitrogen gas (heater temperature 300 °C) was maintained at 7 psig. In TOFMS, the fragmentor-, skimmer-, and Oct RFV voltage was set at 75 V, 50 V, and 500 V, respectively. Automatic recalibration of each acquired spectrum was performed using reference masses of reference standards. The ^13^C isotopic ion of a protonated methanol dimer ([2MeOH + H]^+^, m/z 66.0631) and Hexakis(2,2-difluoroethoxy)phosphazene ([M + H]^+^, m/z 622.0290) provided the lock mass for exact mass measurements (Satoh et al., 2017).

#### Anionic metabolite analysis

A commercially available COSMO( +) (chemically coated with cationic polymer) capillary (50 mm i.d. × 105 cm total length) (Nacalai Tesque, Kyoto, Japan) was used with 50 mM ammonium acetate solution (pH 8.5) as the electrolyte (Satoh et al., 2017; Soga et al., 2009). Sample solution (30 nl) was injected at 50 mbar for 30 s and -30 kV of voltage was applied. Ammonium acetate (5 mM) in 50% methanol–water (v/v) containing 0.01 mM Hexakis(2,2-difluoroethoxy)phosphazene was delivered as the sheath liquid at 10 μl/min. ESI-TOFMS was conducted in the negative ion mode; the capillary voltage was set at 3500 V. For TOFMS, the fragmentor-, skimmer-, and Oct RFV voltage was set at 100 V, 50 V, and 500 V, respectively. Automatic recalibration of each acquired spectrum was performed using reference masses of reference standards, i.e., ^13^C isotopic ion of deprotonated acetic acid dimer ([2CH_3_COOH-H]^−^, m/z 120.0384), and Hexakis + deprotonated acetic acid (m/z 680.03554) provided the lock mass for exact mass measurements.

### FDG PET/CT imaging and analysis

FDG was manufactured using an automated synthesis system with an HM-18 cyclotron (QUPID; Sumitomo Heavy Industries Ltd, Tokyo, Japan).

All acquisitions were performed using a Biograph mCT 64-slice PET/CT scanner (Siemens Healthcare, Erlangen, Germany) with an axial field of view of 21.6 cm. The patients fasted for at least 5 h prior to FDG administration, and a normal glucose level in the peripheral blood was confirmed prior to injection. Emission data were obtained 90 min after the intravenous injection of FDG (5 MBq/kg) from the midcranium to the proximal thighs (2 min per bed position). Unenhanced low-dose CT of the same area was performed for attenuation correction and image fusion. PET data were reconstructed using a Gaussian filter with an ordered subset expectation maximization algorithm, incorporating correction with the point-spread function and time-of-flight model (two iterations, 21 subsets).

A board-certified nuclear medicine physician performed the volume-based analyses using syngo.via (Siemens Healthcare, Erlangen, Germany). The volume of interest of the primary tumor was selected using a threshold of 40% of the SUVmax; thereafter, the MTV (defined as lesion volume with uptake) was calculated. The MTV was dichotomized by the median value, and the tumors were divided into low-MTV and high-MTV tumors. The SUVmax, mean SUV (SUVmean), and peak SUV (SUVpeak; a 1.2-cm-diameter sphere positioned to maximize the mean value) were also calculated.

### Statistical analyses

Data were analyzed using SPSS statistical software (version 28; IBM). Metabolomics data were compared between the low-MTV and high-MTV tumors using the Mann–Whitney *U* test. Differences in MTV among TNM classification (stages I–IV) and tumor location (right colon, left colon, and rectum) were compared using the Kruskal–Wallis test. The Bonferroni-corrected Mann–Whitney test was used for the post hoc analysis. Spearman’s rank correlation coefficient was used to determine the degree of correlation between the MTV and patient’s age. A *P-value* of less than 0.05 was considered statistically significant.

## Results

**Fig. 1:**
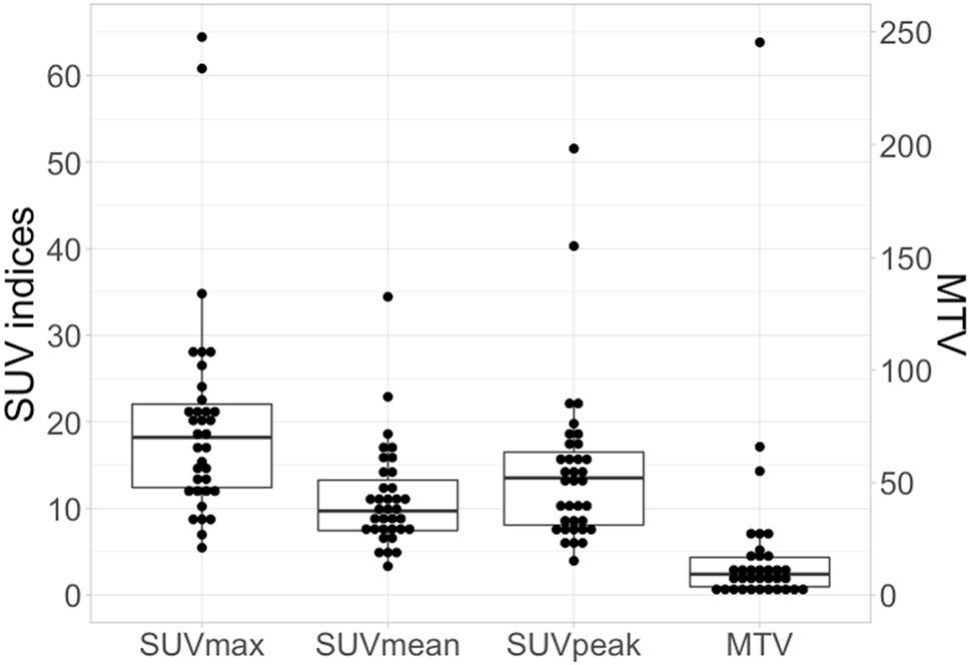
The distribution of SUV indices and MTV of the 35 tumor samples. Box plots show median, 25th percentile, and 75th percentile of data, with minimum and maximum represented by whiskers

Figure 1 shows the distribution of SUV indices and the MTV of the 35 tumor samples.

More than 500 ionic metabolites were measured using CE-TOFMS; however, we analyzed 120 metabolites detected in > 50% of the samples because some of these metabolites were only detected in a few samples.

There was no significant difference in the MTV among TNM classification, tumor location, and patient’s age. Tumors (n = 35) were divided into low-MTV and high-MTV tumors based on the median value of MTV (9.26 cm^3^).

**Fig. 2:**
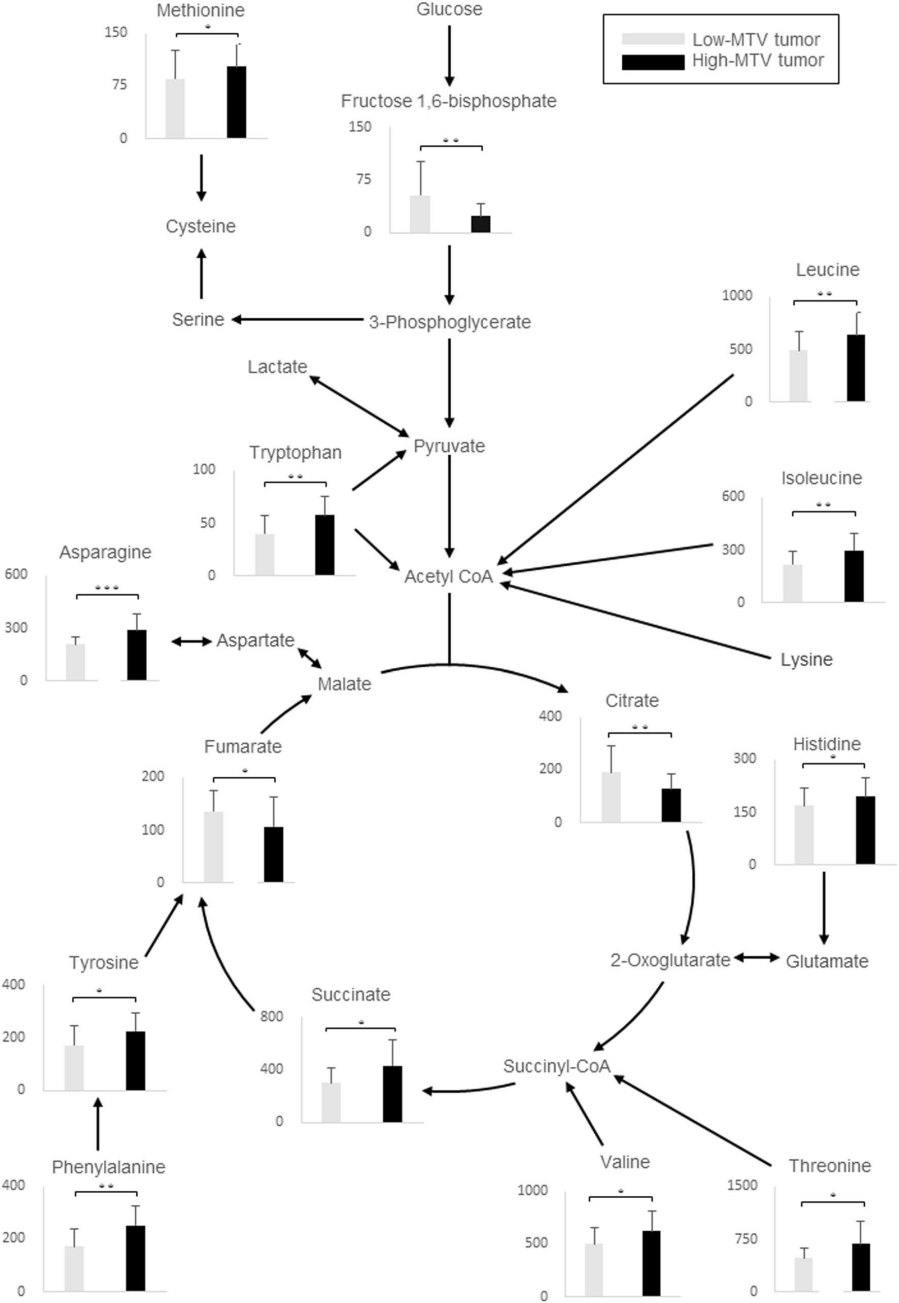
Metabolite concentrations in colorectal cancer tissue superimposed on a metabolic pathway map that included glycolysis, TCA cycle, and amino acids. Columns, average concentration (nmol/g tissue) of tumor tissues based on low-FDG and high-FDG metabolic tumor volume; bars, SD. All *P* values were evaluated using the Mann–Whitney *U* test. *, *P* < 0.05; **, *P* < 0.01; ***, *P* < 0.001

Significant differences were observed in 31 metabolites between the low-MTV and high-MTV tumors (Table 1). The levels glycolysis-related metabolites, except fructose 1,6-bisphosphate, were not different between the low-MTV and high-MTV tumors. The lactate level was not different between the low-MTV and high-MTV tumors. The level of component of the initial part of the TCA cycle, citrate, was significantly lower in the high-MTV tumor than in the low-MTV tumor. The TCA intermediate succinate level was significantly higher in the high-MTV tumor than in the low-MTV tumor. In contrast, the TCA intermediate fumarate level was significantly lower in the high-MTV tumor than in the low-MTV tumor (Fig. 2). The levels of many amino acids were significantly higher in the high-MTV tumor than in the low-MTV tumor. Some purine and pyrimidine metabolism levels were significantly lower in the high-MTV tumor than in the low-MTV tumor.

**Table 1:** The list of significantly changed metabolites (*P* < 0.05) between low-MTV and high-MTV tumors based on the median value

Metabolites	Low-MTV	High-MTV	*P* value
Fructose 1,6-bisphosphate	54 ± 47	24 ± 17	0.004
Methionine	85 ± 40	102 ± 32	0.035
Citrate	192 ± 100	128 ± 54	0.008
Succinate	301 ± 115	433 ± 192	0.02
Fumarate	136 ± 38	108 ± 57	0.027
Ethanolamine phosphate	4683 ± 1446	3234 ± 1101	0.011
N-acetylaspartate	43 ± 12	34 ± 17	0.027
Cytidine monophosphate	23 ± 10	14 ± 9.1	0.006
Uridine monophosphate	326 ± 197	170 ± 151	0.02
Inosine monophosphate	249 ± 213	108 ± 124	0.003
Adenosine diphosphate	487 ± 246	290 ± 122	0.013
Adenosine triphosphate	185 ± 175	83 ± 79	0.029
Histamine	73 ± 59	37 ± 34	0.019
Betaine	169 ± 104	89 ± 66	< .001
Valine	500 ± 160	623 ± 193	0.015
Threonine	480 ± 147	681 ± 330	0.011
Isoleucine	220 ± 76	298 ± 100	0.009
Leucine	489 ± 178	637 ± 213	0.006
Asparagine	208 ± 46	287 ± 96	< .001
Hypoxanthine	425 ± 217	627 ± 323	0.035
Histidine	169 ± 49	194 ± 56	0.035
Phenylalanine	172 ± 64	250 ± 74	0.002
Tyrosine	171 ± 73	224 ± 71	0.01
O-acetylcarnitine	263 ± 197	138 ± 57	0.003
Tryptophan	40 ± 17	58 ± 17	0.001
6-Phosphogluconate	21 ± 17	12 ± 14	0.035
N-acetylglucosamine 6-phosphate	25 ± 11	41 ± 20	0.01
S-adenosylhomocysteine	14 ± 6.3	20 ± 8.2	0.008
Hexanoate	9.5 ± 12	33 ± 72	0.018
1-Methylhistamine	4.6 ± 2.5	2.8 ± 2.2	0.025
NADPH	26 ± 18	16 ± 14	0.045

Data are givens as mean ± standard deviation. *MTV* metabolic tumor volume

## Discussion

Metabolomics is a scientific research involving metabolites, small-molecule substrates, intermediates, and products of cell metabolism. Therefore, metabolomics provides a direct functional readout of physiological state (Hollywood et al., 2006). The present study focused on the association between the FDG MTV and metabolomics data in surgically resected tumor tissues from 35 CRCs. To the best of our knowledge, no studies have reported the association between FDG PET/CT and metabolomics data in patients with CRC.

Hirayama et al. compared the metabolite levels in normal and tumor tissues obtained from patients with colon cancer (Hirayama et al., 2009). High lactate and glycolytic intermediate concentrations were observed in colon tumor tissues, which indicated enhanced glycolysis and, thus, confirmed the Warburg effect (Hirayama et al., 2009). In the present study, the levels of most glycolytic metabolites and the lactate levels were not significantly different between the low-MTV and high-MTV tumors. Glycolysis may be enhanced in colon cancer, regardless of high FDG metabolism.

The level of component of the initial part of the TCA cycle, citrate, was significantly lower in the high-MTV tumor than in the low-MTV tumor. Succinate concentration was significantly higher in the high-MTV tumor than in the low-MTV tumor. In contrast, fumarate concentration was significantly lower in the high-MTV tumor than in the low-MTV tumor. It remains unclear what causes the accumulation of succinate in high-MTV tumor, despite low citrate and fumarate levels. Tumors with high FDG metabolism in CRC may obtain more energy by using a reverse reaction of the TCA cycle because it is known that some parasites and bacteria synthesize ATP without oxygen using a reverse reaction of succinate dehydrogenase and produce succinate as a by-product (Hirayama et al., 2009; Kita et al., 2002). However, the capability of mammalian cells to use a reverse reaction of the TCA cycle has not been confirmed. Therefore, further research is needed to address this issue.

The levels of many amino acids were significantly higher in the high-MTV tumor than in the low-MTV tumor. Hence, the high-MTV tumor compared to low-MTV tumor might generate energy using amino acids. Moreover, as cancer cells are known to use amino acids as energy sources, the availability of amino acids is crucial for cell proliferation (Argilés & Azcón-Bieto, 1988).

Some purine and pyrimidine metabolism levels were significantly lower in the high-MTV tumor than in the low-MTV tumor. Hypoxic stress has been shown to reduce purine and pyrimidine pools (Hisanaga et al., 1986). Tumors with high FDG metabolism may have more hypoxic stress than those with low FDG metabolism.

Metabolomics has been applied to identify metabolic alterations in CRC that may provide clinically useful biomarkers (Zhang et al., 2014). There is an urgent need for biomarkers to make early diagnosis of CRC, assess the therapeutic effect, and predict prognosis. In a study by Ong et al. on the metabolomic profiles in colonic tissues including tumor, polyps and adjacent matched normal mucosa, various amino acids and lipids in the polyps and tumors were found to be elevated, suggesting higher energy requirements for increased cell proliferation (Ong et al., 2010). In contrast, significant depletion of glucose and inositol in polyps showed that glycolysis may be critical in early CRC. Leichtle et al. performed serum metabolic profiling in CRC and showed multiple significant disease-associated alterations in the amino acid profile (Leichtle et al., 2012). However, even if studies on metabolomics reveal useful biomarkers, it is difficult to identify the lesion site. It is possible to identify the lesion site by PET examination. Especially, in addition to FDG that reflects glucose metabolism, PET probes that reflect characteristic metabolism in cancer, such as amino acid metabolism and nucleic acid metabolism, have been developed (Groves et al., 2007). The combination of metabolomics and PET has a great potential for early detection of CRC, therapeutic monitoring, and predicting prognosis.

The present study is the first to investigate the relationship between FDG PET/CT and metabolomics data in patients with newly diagnosed CRC.　A limitation of this study was that the results obtained were based on a relatively small number of patients at a single institution. The study cohort was very heterogeneous concerning age, TNM classification, and tumor location. In the future, network analysis will probably overcome the problem of small numbers in each subgroup. The FDG metabolic indices in this patient population were also heterogeneous. We did not perform histopathological analyses such as measurement of the percentage infiltration or Ki-67 index on the mirror sample compared to the sample used for metabolomics. Large variations were reported in metabolome analysis, possibly because of the small sample size, high intratumor heterogeneity, and site-specific differences in tissue structures (Ohta et al., 2010). Therefore, further studies with a larger number of patients are required.

## Conclusion

These preliminary findings suggest that tumors with high FDG metabolism in CRC cases may obtain more energy by using a reverse reaction of the TCA cycle and amino acid metabolism. Our data from a small patient population do not support a clear-cut relationship between FDG PET/CT and metabolomics data. Further studies with a greater number of patients will help clarify this relationship.

## Data Availability

Data that support the findings of this study are available from the corresponding author, [Y.Y], upon reasonable request.
